# ResearchMaps.org for integrating and planning research

**DOI:** 10.1371/journal.pone.0195271

**Published:** 2018-05-03

**Authors:** Nicholas J. Matiasz, Justin Wood, Pranay Doshi, William Speier, Barry Beckemeyer, Wei Wang, William Hsu, Alcino J. Silva

**Affiliations:** 1 Department of Neurobiology, University of California Los Angeles, Los Angeles, California, United States of America; 2 Medical Imaging Informatics, Department of Radiological Sciences, University of California Los Angeles, Los Angeles, California, United States of America; 3 Department of Bioengineering, University of California Los Angeles, Los Angeles, CA, United States of America; 4 Department of Computer Science, University of California Los Angeles, Los Angeles, CA, United States of America; 5 Department of Neurosurgery, University of California Los Angeles, Los Angeles, CA, United States of America; 6 Department of Psychiatry & Biobehavioral Sciences, University of California Los Angeles, Los Angeles, California, United States of America; 7 Department of Psychology, University of California Los Angeles, Los Angeles, California, United States of America; 8 Integrative Center for Learning and Memory, University of California Los Angeles, Los Angeles, California, United States of America; 9 Brain Research Institute, University of California Los Angeles, Los Angeles, California, United States of America; Centrum Wiskunde & Informatica (CWI) & Netherlands Institute for Systems Biology, NETHERLANDS

## Abstract

To plan experiments, a biologist needs to evaluate a growing set of empirical findings and hypothetical assertions from diverse fields that use increasingly complex techniques. To address this problem, we operationalized principles (e.g., convergence and consistency) that biologists use to test causal relations and evaluate experimental evidence. With the framework we derived, we then created a free, open-source web application that allows biologists to create *research maps*, graph-based representations of empirical evidence and hypothetical assertions found in research articles, reviews, and other sources. With our *ResearchMaps* web application, biologists can systematically reason through the research that is most important to them, as well as evaluate and plan experiments with a breadth and precision that are unlikely without such a tool.

## Introduction

Information in biology falls into at least two categories: (1) the information that individual biologists curate from articles they read, and (2) the vast body of other information that biologists can access, at least in principle, through resources like PubMed. Most informatics tools target the second category: the literature’s accelerating growth makes it exceedingly impractical for biologists to find all the information that is relevant to their work. But even within the first category, it is ever more difficult for biologists to synthesize the information that they personally curate. Part of this challenge is caused by the increasing complexity of biological research.

Individual biologists must now keep track of empirical findings and hypothetical assertions from diverse fields that use a growing number of sophisticated techniques. Perhaps an even greater problem is that biologists are tasked with synthesizing this complex web of information with little help from machines. Thus, biologists could benefit from methods to help them track the information they deem critical for integrating and planning experiments. Given the unmatched ability of computers to index, retrieve, and process information, biologists could benefit enormously from a software tool capable of helping them to track and reason through causal assertions; such a tool could help biologists to synthesize empirical findings and plan future experiments.

Building on our previous discussions of these general concepts [1–3], we introduce an updated *research map* representation and an accompanying web application, *ResearchMaps* (http://researchmaps.org/), designed to help biologists integrate and plan experiments. A research map graphically represents hypothetical assertions and empirical findings. To weigh the evidence encoded in a research map, we present a novel Bayesian calculus of evidence that allows researchers to formally synthesize empirical results. This Bayesian approach expresses integration principles, including convergence and consistency, commonly used by many biologists to judge the strength of causal assertions. Thus, our goal with research maps was not to build another *ontology* but rather to formalize aspects of biologists’ *epistemology* [3].

Biologists traditionally find research summaries in reviews and opinion articles. Although these articles are useful, they have clear limitations: they are not dynamically updated; it is cumbersome to personalize them; and they usually reflect the state of a field as it existed at least one to two years before the publication date. These limitations are particularly a problem in rapidly changing fields like neuroscience. By comparison, digital lab notebooks are extremely useful for tracking and sharing experiments and findings between collaborators, but they are not designed to track large amounts of causal information in a representation that facilitates evidence synthesis, knowledge discovery, or causal reasoning.

Existing representations such as Knowledge Engineering from Experimental Design (KEfED) provide a way to model experimental procedures and findings in a detailed and machine-readable manner [4]. Here, we present a complementary approach for representing and querying high-level assertions that characterize connections among phenomena. Moreover, formalisms such as probabilistic graphical models (e.g., Bayesian networks) have been shown to be effective at conveying relations among biological phenomena using a graph structure, as they compactly encode the joint probability distribution across variables [5]. However, conditional probabilities are often missing in reports of experiments designed to test causal assertions. Pathway analysis tools such as BioCarta [6] and Ingenuity Pathways Analysis (QIAGEN Redwood City, Redwood City, CA, USA) provide graphical representations of possible causal connections, but they do not keep track of the classes of experiments carried out to arrive at those conclusions, and they are usually restricted to specific domains of biological phenomena (e.g., molecular interactions).

The research-map framework shares some similarities with the recent WatsonPaths™ system, in which an assertion graph is constructed to reason through medical information [7]. Unlike a WatsonPaths assertion graph, a research map reflects a personally curated knowledge representation for a specific domain or sequence of experiments. Thus, a research map requires no training examples or external knowledge bases to be created.

## Results

### Research maps

**Fig 1:**
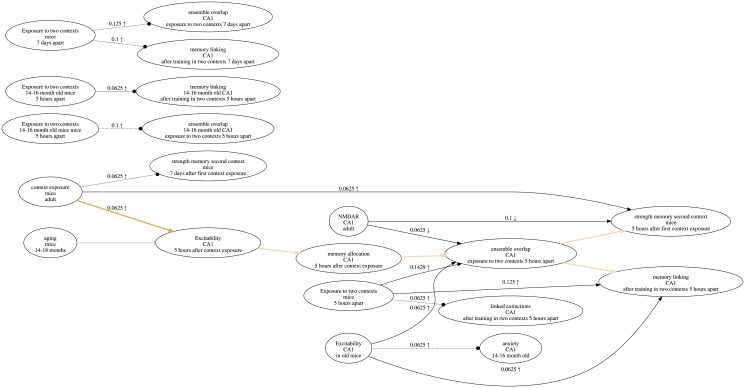
Research map of the results in a published article [8]. Each node in a research map has three properties: What (top), Where (middle), and When (bottom). Nodes are connected by edges that represent relations: Excitatory (sharp arrowhead), Inhibitory (blunt arrowhead), and No-connection (dotted line, circular arrowhead). Each empirical edge also has a score that reflects the amount of evidence represented, as well as symbols that reflect the experiment classes recorded for that edge. Scores and experiment symbols are not assigned to hypothetical edges. Users can highlight edges that reflect the main idea(s) discussed in the article, so that they are more apparent. In cases where no one relation has received dominant evidence, the corresponding edge is represented by a diamond arrowhead and is not assigned a score.

A research map is a directed graph that represents information concerning possible causal relations between biological phenomena [1]. Each node in the graph represents the identity and properties of a biological phenomenon, and each directed edge—from an *Agent* node to a *Target* node—represents a relation between phenomena (e.g., *A*→*B*). In an experiment, an Agent is either intervened on or observed; this Agent may or may not act on another phenomenon, the Target, which is measured in the experiment. An Agent for one edge can be a Target for another. The key concepts captured in research maps reflect common epistemic practices in many fields of biology, represented in areas as diverse as neuroscience, development, immunology, and cancer. Thus, research maps should be useful to represent information in these and other areas of biology. See Fig 1 for an example of a research map of a published article [8].

### The framework of research maps

In research maps, biological experiments are categorized according to a hierarchical framework [9]. We propose that experiments in many fields of biology can be classified into three general classes: (1) *Identity Experiments* attempt to identify phenomena and their properties; (2) *Connection Experiments*, the subject of research maps, test causal hypotheses; and (3) *Tool Development Experiments* develop and evaluate tools for performing Identity and Connection Experiments.

Within the class of Connection Experiments, we propose that there are four subclasses of experiments used to test a hypothesized connection between an Agent *A* and a Target *B*: (1) *Positive Intervention*, (2) *Negative Intervention*, (3) *Positive Non-intervention*, and (4) *Negative Non-intervention*. In a Positive Intervention experiment, the quantity or probability of the Agent *A* is increased, and the change (or lack of change) in Target *B* is measured. For example, to determine whether the activity of cell type *A* affects memory *B*, one could increase the activity of cell type *A* and then study the impact on memory *B*. In this case, the activity of cell type *A* is actively increased via an intervention—for instance, optogenetically.

A Negative Intervention experiment decreases the quantity or probability of *A* and measures *B*. For example, we could study how memory *B* is effected by a manipulation that inhibits cell type *A*. Positive and Negative Intervention experiments thus complement each other: the two use different approaches to probe the strength of the hypothesized connection between *A* and *B*.

Using only Positive and Negative Intervention experiments raises a number of problems that could confound the interpretation of those experiments. For example, such experiments always impose a change in an Agent *A* with methods that could have unintended effects. Therefore, any change observed in Target *B* may not necessarily result from a causal connection between *A* and *B* that is observable under specific conditions (e.g., during a spatial learning task); the change in *B* could instead be caused by experimental side effects of artificially intervening on *A*. The experimental process of intervening on *A* may inadvertently affect another phenomenon, *C*, even if *C* is not normally affected by *A* outside of the experimental setting. Although *C* may be the true cause of *B*, it may appear to the experimenter, who is oblivious to *C*’s involvement, that *A* causes *B*. This possibility demonstrates the need for Non-intervention experiments to complement Positive and Negative Interventions.

A Non-intervention experiment measures *A* and *B* without intervening on either. In a Positive Non-intervention experiment, the quantity or probability of *A* is observed to increase, and the change (or lack of change) in *B* is measured. In a Negative Non-intervention experiment, the quantity or probability of *A* is observed to decrease, and *B* is measured. These experiments help us to learn whether the relation between *A* and *B* identified by Intervention experiments exists outside of the experimental setting used to intervene on *A*. Without Non-intervention experiments, it is difficult to be sure that experimental results are not mere artifacts caused by the interventions used to change *A*. In many fields of biology, Non-intervention experiments alone are usually judged to be insufficient to determine whether two phenomena are causally connected, as they are thought to merely document the correlation between these phenomena. However, elegant methods have been developed to identify specific causal structures solely from patterns of correlations derived from observational (i.e., non-interventional) data [10].

From the four classes of experiments described above, we can glean evidence for three types of relations between phenomena. A relation between an Agent and a Target is defined as *Excitatory* when an increase in the Agent leads to an increase in the Target, or a decrease in the Agent leads to a decrease in the Target. In an Excitatory relation, a Positive Intervention experiment would result in an increase in the Target, and a Negative Intervention experiment would result in a decrease of the Target. In an *Inhibitory* relation, an increase in the Agent leads to a decrease in the Target, while a decrease in the Agent leads to an increase in the Target. When changes in the Agent fail to affect the Target, there is evidence for the absence of a connection between the two phenomena. In this last case, although the Agent and Target do not appear to be connected, this independence is represented explicitly with a relation denoted as *No-connection*.

### Rules of integration

In biology—and in research maps—a key approach to determine the reliability of results and the usefulness of hypotheses is to look for convergence and consistency in a set of findings. For instance, we can ask whether *A* reliably affects *B* or whether *A* and *B* are consistently independent of each other. We refer to the process that attempts to combine a series of experimental results as *Integration* [9].

Integration methods determine the strength of the evidence for a particular connection, which is quantified and expressed as a score for a particular edge in a research map. The evidential strength of a connection is not to be confused with the magnitude of the causal effect that *A* has on *B*, where *A* may be one of many possible causes of *B*. Integration methods include (but are not limited to) *Convergence Analysis* and *Consistency Analysis* [9]. By gauging the extent to which evidence is convergent and consistent, these Integration methods help to distinguish hypotheses with strong support from those with weak support. The principles of convergence and consistency are thus used for instantiating and scoring empirical edges in research maps.

Convergence Analysis assesses whether the outcomes of the different kinds of Connection Experiments (Positive and Negative Interventions, and Positive and Negative Non-interventions) are consistent with each other—i.e., whether they support a single connection type (either Excitatory, Inhibitory or No-connection). Suppose we find that optogenetically inhibiting cell type *A* is associated with a deficit in spatial learning. Suppose also that enhancing the activity of cell type *A* enhances the same form of learning. If we also found that cell type *A* is activated during spatial learning, and that this cell type is inactive when the animal is not learning, then our combined results would make a compelling argument that the activation of cell type *A* is causally connected to spatial learning. This convergence between these four classes of experiments would yield a relatively high score for the Excitatory connection in a research map representing the relation between cell type *A* and spatial learning. On the other hand, contradictions among the data would lower the score of the connection. Convergence Analysis thus encompasses the notions that multiple lines of evidence are preferable to one, and that different experiment classes make unique contributions to testing the reliability of a hypothesized connection between two phenomena.

In addition to gauging the convergence of experimental results across multiple classes of experiments, it is also important to gauge the consistency of experimental results within each class of experiment. For this purpose, Consistency Analysis assesses whether experimental results are reproducible. For example, we might ask whether different kinds of Positive Interventions on the activity of cell type *A* (e.g., chemogenetic and optogenetic) always result in an enhancement of spatial learning. This question can refer to multiple iterations of the exact same experiment, or to a set of experiments that are similar in principle—e.g., two Positive Interventions of receptor *A*, one chemogenetic and the other optogenetic, that test two different forms of spatial learning.

### Calculating scores

To convey the amount of evidence for a particular empirical edge in a research map, a score for the edge is calculated using an algorithm based on the Integration methods above. These methods reflect epistemological rules and commonsense intuitions found in fields that use molecular and cellular approaches to biological problems, including neurobiology, biochemistry, cell biology, and physiology. In designing an approach to scoring such evidence, we strove to express quantitatively the following axioms: the principles of (1) convergence and (2) consistency, as described above; (3) the principle that convergence carries greater epistemological weight than consistency; and (4) the principle that we have no *a priori* reason to prefer one class of experiment to another when aggregating evidence. (In areas of science where one type of experiment is favored over others for technical reasons, our approach allows for a non-uniform weighting of evidence from different experiment classes.) There are other axioms used in science that have not been expressed in the scoring algorithm of research maps [9] because we see them to be secondary to the ones above.

The central idea of our scoring approach is that convergent and consistent results increase the score of an edge, while conflicting results decrease the score. Each score falls in the range (0, 1), and each experiment class (Positive Intervention, Negative Intervention, Positive Non-intervention, and Negative Non-intervention) contributes an amount in the range (0, 0.25) to the overall score. Multiple experiments of the same kind contribute progressively smaller scores to the edge. As experiments are recorded, a Bayesian approach is used to update the degrees of belief attributed to each type of relation. The scores thus reflect an approach for gauging the strength of the convergent and consistent evidence supporting a given connection; their semantics are derived not from their absolute values but from their relative values. In addition to p-values from statistical tests and associated meta-analyses, this scoring method could conceivably be used to evaluate the strength of evidence across various types of experiments testing a single causal assertion.

The score for an edge in a research map is calculated as follows. Let *C* = {↑, ⌀^↑^, ⌀^↓^, ↓} denote the set of all experiment classes, where *c* = ↑ denotes the class Positive Intervention; *C* = ⌀^↑^ denotes the class Positive Non-intervention; *C* = ⌀^↓^ denotes the class Negative Non-intervention; and *c* = ↓ denotes the class Negative Intervention. Let R={E,N,I} denote the set of relations that can exist between two phenomena and for which an experiment can provide evidence, where E denotes an Excitatory relation; N denotes a No-connection relation; and I denotes an Inhibitory relation. Thus, an experiment of class *C* ∈ {↑, ⌀^↑^, ⌀^↓^, ↓} can yield evidence in support of relation r∈{E,N,I}.

Let $\alpha_{c}=(\alpha_{c,E},\alpha_{c,N},\alpha_{c,I})$; let $\theta_{c}=(\theta_{c,E},\theta_{c,N},\theta_{c,I})$, and let $x_{c}=(x_{c,E},x_{c,N},x_{c,I})$, where

$$\begin{array}{c} (\theta_{c,E},\theta_{c,N},\theta_{c,I})∼\text{Dir}\left(\alpha_{c,E},\alpha_{c,N},\alpha_{c,I}\right)\;, \end{array}$$ (1)

$$\begin{array}{c} (x_{c,E},x_{c,N},x_{c,I})∼\text{Mult}\left(\theta_{c,E},\theta_{c,N},\theta_{c,I},n_{c}\right)\;. \end{array}$$ (2)

**Fig 2:**
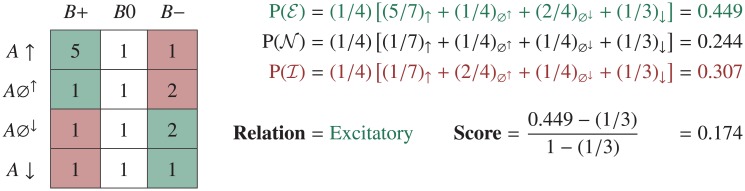
A shorthand method for calculating the score of an edge in a research map. A table representing the model space of experiments is instantiated with a pseudocount of one (a form of Laplace smoothing). The symbols along the left indicate the classes of experiments involving an Agent, *A*: Positive Intervention (*A* ↑), Positive Non-intervention (*A*⌀^↑^), Negative Non-intervention (*A*⌀^↓^), and Negative Intervention (*A* ↓). The symbols along the top indicate the results recorded in a Target, *B*: increase (*B*+), no change (*B*0), and decrease (*B*−). This particular instantiation of the scoring table encodes four (5 − 1) Positive Interventions that caused the Target to increase, one (2 − 1) Positive Non-intervention that caused the Target to decrease, and one (2 − 1) Negative Non-intervention that caused the Target to decrease. There are thus five experiments suggesting an Excitatory relation (green regions), and one experiment suggesting an Inhibitory relation (red region).

Here, *α*_*c*,*r*_ is the prior weight given to relation *r* supported by experiments of class *c*; *θ*_*c*,*r*_ is the probability that the next experiment of class *c* will yield evidence in support of relation *r*; *x*_*c*,*r*_ is the number of experiments of class *c* that have yielded evidence in support of relation *r*, and *n*_*c*_ is the number of experiments of class *c* that have been performed. For each class of experiment *c*, we can define ***x***_*c*_ (compare to the table in Fig 2):

$$x_{↑}=[x_{↑,E},x_{↑,N},x_{↑,I}]\;,$$ (3)

$$x_{{⌀}^{↑}}=[x_{{⌀}^{↑},E},x_{{⌀}^{↑},N},x_{{⌀}^{↑},I}]\;,$$ (4)

$$x_{{⌀}^{↓}}=[x_{{⌀}^{↓},E},x_{{⌀}^{↓},N},x_{{⌀}^{↓},I}]\;,$$ (5)

$$x_{↓}=[x_{↓,E},x_{↓,N},x_{↓,I}]\;.$$ (6)

The score of an edge is based on the values of *θ*_*c*_ for each of the experiment classes, which are updated as additional experiments are recorded, thereby changing the values of ***x***_*c*_. We are thus interested in estimating each ***θ***_*c*_ in light of the evidence represented by each ***x***_*c*_. Applying Bayes theorem yields

$$p(\theta_{c}|x_{c},\alpha_{c})\propto p\left(x_{c}|\theta_{c}\right)p\left(\theta_{c}|\alpha_{c}\right)\;,$$ (7)

$$\begin{array}{cc} & \propto \theta_{c,E}^{\alpha_{c,E}+x_{c,E}-1}\theta_{c,N}^{\alpha_{c,N}+x_{c,N}-1}\theta_{c,I}^{\alpha_{c,I}+x_{c,I}-1}\;, \end{array}$$ (8)

The posterior distribution is in the form of a Dirichlet distribution, so we have that

$$\begin{array}{c} \theta_{c}|x_{c},\alpha_{c}∼Dir\left(\alpha_{c}+x_{c}\right)\;. \end{array}$$ (9)

The expected value of this distribution is thus expressed as

$$\begin{array}{c} E\left[\theta_{c,r}|x_{c},\alpha_{c}\right]=\frac{\alpha_{c,r}+x_{c,r}}{\sum_{r}\alpha_{c,r}+n_{c}}\;. \end{array}$$ (10)

If *α*_*c*,*r*_ = 1 for all *c* and *r*, the above expression becomes

$$\begin{array}{c} E\left[\theta_{c,r}|x_{c},\alpha_{c,r}=1\right]=\frac{1+x_{c,r}}{\left|R\right|+n_{c}}\;, \end{array}$$ (11)

which is an implementation of Laplace (add-one) smoothing.

In the absence of evidence (i.e., before any experiments are performed), *x*_*c*,*r*_ = 0 for all *c*, *r*. We denote this state by *θ*_*o*_:

$$\begin{array}{c} \theta_{o}=E\left[\theta_{c,r}|x_{c}=(0,0,0),\alpha_{c,r}=1\right]=\frac{1}{\left|R\right|}=\frac{1}{3}\;. \end{array}$$ (12)

Let $\bar{\theta }$ denote the set of mean *r*-components across all experiment classes (an expression of convergence):

$$\bar{\theta }=\frac{1}{\left|C\right|}[\sum_{c}E\left[\theta_{c,E}|x_{c},\alpha_{c,E}=1\right],\sum_{c}E\left[\theta_{c,N}|x_{c},\alpha_{c,N}=1\right],\sum_{c}E\left[\theta_{c,I}|x_{c},\alpha_{c,I}=1\right]]\;.$$ (13)

The relation assigned to the research-map edge is the relation with the largest component in $\bar{\theta }$:

$$\begin{array}{c} \underset{r}{\mathrm{argmax}}\;{\bar{\theta }}_{r}\;. \end{array}$$ (14)

The score assigned to the research-map edge is

$$\begin{array}{c} \frac{\max\bar{\theta }-\theta_{o}}{1-\theta_{o}}\;, \end{array}$$ (15)

where $\max\bar{\theta }$ denotes the largest component of $\bar{\theta }$. In cases where two or more components of $\bar{\theta }$ are equal, neither a relation nor a score is assigned to the edge.

**Fig 3:**
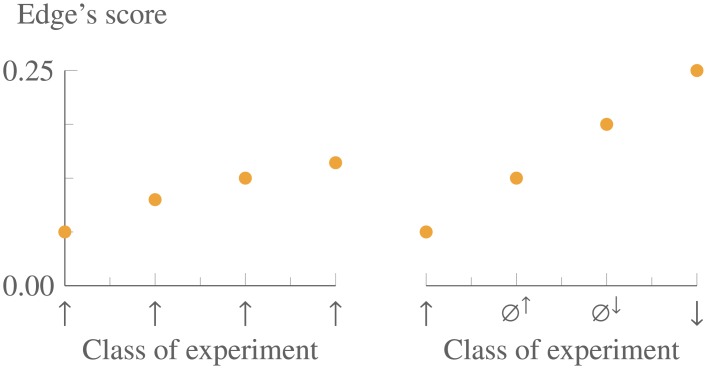
The growth of an edge’s score due only to consistency (left) and due to convergence (right). These plots show how the score of a research-map edge increases with each subsequent experiment (all with agreeing results), due to the principle of consistency (left) and due to the principle of convergence (right). The plot on the left represents repeated iterations of the same class of experiment (e.g., Positive Intervention) with consistent results. The plot on the right represents multiple iterations of experiments in which, at each iteration, one of the least-represented classes of experiments was performed, leading to consistent results. These two plots express an axiom of research maps: the principle of convergence carries greater epistemological weight than the principle of consistency.

See Fig 2 for a depiction of a shorthand calculation of an edge’s score. See Fig 3 for plots of how the score of an edge increases with each subsequent experiment due to the principles of consistency and convergence.

It is worth noting that the scores derived from the above scoring algorithm, which is based on Bayesian principles, closely resemble those derived from a heuristic scoring approach used in early versions of research maps, which expressed scientists’ intuitions regarding the integration of evidence [2]. See S1 Fig for a comparison of these two scoring approaches; see Sed S2 Fig for the derivation of the earlier heuristic scoring approach.

### A scoring example

**Fig 4:**
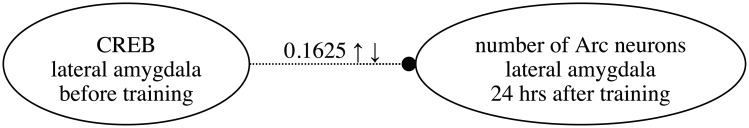
An example of an edge in a research map. This research map encodes three experiments—two Positive Interventions (↑) and one Negative Intervention (↓)—involving CREB and the number of Arc neurons. This map is part of a larger one that is discussed below.

To develop an intuition for the above scoring approach, consider the following example, which uses the experiments involving CREB and the number of Arc neurons that are depicted in Fig 4. In this research map, the edge connecting these two nodes represents three experiments: two Positive Interventions of CREB resulting in no change in the number of Arc neurons, and one Negative Intervention of CREB, again resulting in no change. Together, these three experiments provide evidence for a No-connection edge between the two nodes. Before any of these experiments were performed, ***θ***_*c*_ was uniform for all *c*. After the first experiment, in which a Positive Intervention produced no change in the Target, ***θ***_*c*_ = (0.25, 0.50, 0.25) and the score of the edge was 0.0625. After the second Positive Intervention (with the same result as the first), the score of the edge became 0.1000.

The first Positive Intervention thus changed the score by 0.0625, while the second experiment changed the score by 0.0375. These two changes in the score demonstrate a commonsense intuition regarding evidence that is expressed quantitatively by the scoring algorithm: each subsequent experiment that yields consistent results increases the score, albeit by an amount that is less than the amount contributed by the previous consistent experiment.

After the third experiment, in which a previously unrepresented experiment class (Negative Intervention) yielded a consistent result (no change), the score increased to 0.1625, for a net change of 0.0625. This change demonstrates another desirable feature of the scoring algorithm: when consistent results are obtained across multiple experiment classes, each sequence of experiments within a class contributes the same set of decaying amounts to the score, such that results across the four experiment classes are weighted independently of the order in which they were obtained.

If a fourth experiment with conflicting evidence were recorded—for example, a Positive Non-intervention yielding an increase in the Target—the score would drop to 0.1313. Appropriately, the conflicting evidence would undermine the still-dominant evidence that the relation between the two nodes is No-connection. Had this conflicting evidence come from another Positive Intervention, an experiment class already represented in the score, the score would drop to 0.1250. This larger drop (compared to the one incurred for a conflicting Positive Non-intervention) reflects the idea that scientists tend to trust evidence from a particular experiment class to the extent that experiments within this class yield consistent results.

### ResearchMaps

ResearchMaps is a web application that implements the above algorithms and framework, thus allowing users to create, integrate, and interact with research maps. Below we review (1) how we implement research maps in a web interface, and (2) how users can interact with research maps to explore both empirical and hypothetical information.

#### Components of ResearchMaps

In ResearchMaps, an Agent or Target is defined in three complementary ways: *what* the phenomenon is, *where* the phenomenon exists, and *when* the phenomenon acts. ResearchMaps stores this information as three properties for each node: (1) What describes a key identifier of the phenomenon involved (e.g., the name by which the gene, protein, cell, organ, behavior, etc. is known); (2) Where describes the location of the What (e.g., the organ, species, etc.); and (3) When provides temporal information that is critical to the identity of the What (e.g., the time, age, phase, etc.). For example, if the protein neurofibromin is measured in multiple locations, a corresponding research map would include multiple nodes for neurofibromin with different Where properties. This approach is instructive, as neurofibromin could have different biological characteristics in different cellular locations (e.g., excitatory neurons versus inhibitory neurons) or at different stages of development. ResearchMaps displays the What, Where, and When properties on separate lines within each node.

In ResearchMaps, the four experiment classes are represented by symbols above each empirical edge. As given in set *C* above, Positive Interventions are represented by an upward arrow (↑); Negative Interventions are represented by a downward arrow (↓); Positive Non-interventions are represented by the empty set symbol and a superscript upward arrow (⌀^↑^); and Negative Non-interventions are represented by the empty set symbol and a superscript downward arrow (⌀^↓^). Although we have not yet defined a formal representation for experiments involving more than two nodes, ResearchMaps accommodates intervention experiments with two Agents. At the time of this writing, such experiments comprise approximately fourteen percent of the experiments logged. The putative mechanisms underlying the results of these multi-intervention experiments can be visualized using hypothetical edges among the three entities involved (two Agents and one Target); the structure of these hypothetical edges is provided by the user.

ResearchMaps can accommodate information about the statistical test used to establish each finding and its associated p-value. Such information is of course valuable in evaluating experiments; however, as the areas covered by research maps are diverse, and there are no standards as to which statistics are used and how to report them, p-values do not currently affect the score of research-map edges, and they are optionally tracked by each user. See Fig 1 for an example of a research map.

#### Empirical and hypothetical edges

ResearchMaps allows the user to input both empirical and hypothetical edges between any two phenomena (and, by extension, empirical and hypothetical nodes). A hypothetical edge represents a putative connection with no direct experimental evidence. Hypothetical edges are usually implied by empirical edges, and they are often key in interpreting and reporting the results of a research article. Since hypothetical edges do not represent empirical evidence, they are assigned neither scores nor experiment symbols. To visually differentiate hypothetical edges, they are shown in a lighter color and without a score or experiment symbols.

**Fig 5:**
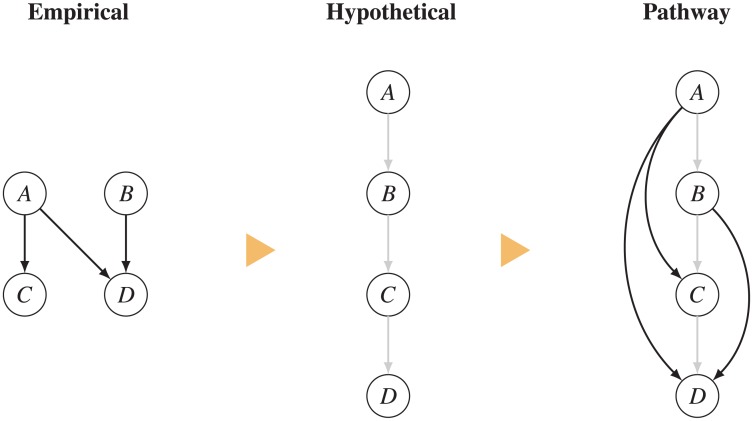
Using hypothetical edges to organize research maps. The example above shows how hypothetical edges (in gray) help to organize empirical edges in a research map, thus framing the empirical results in light of a specific hypothesis.

Beyond allowing users to track various hypotheses, hypothetical edges can also help to structure research maps of empirical evidence, as illustrated with the following example. Consider a signaling pathway (e.g., a biochemical cascade), which we will represent as *A*→*B*→*C*→*D*. Just as hypotheses help to frame and organize the results of research articles, hypothetical edges help to structure and contextualize empirical edges in a research map. For instance, a map that represents the connections *A*→*C*, *A*→*D*, and *B*→*D* (Fig 5) would not explicitly reflect the putative *A*→*B*→*C*→*D* pathway because not all connections in this pathway are part of that map. By including in the resulting map the hypothetical edges *A*→*B*, *B*→*C*, and *C*→*D*, the underlying hypothesis for the experiments carried out is immediately obvious (Fig 5). To further illustrate this point, S3 Fig displays the research map of Fig 1 without its hypothetical edges.

### Generating maps for research articles

**Fig 6:**
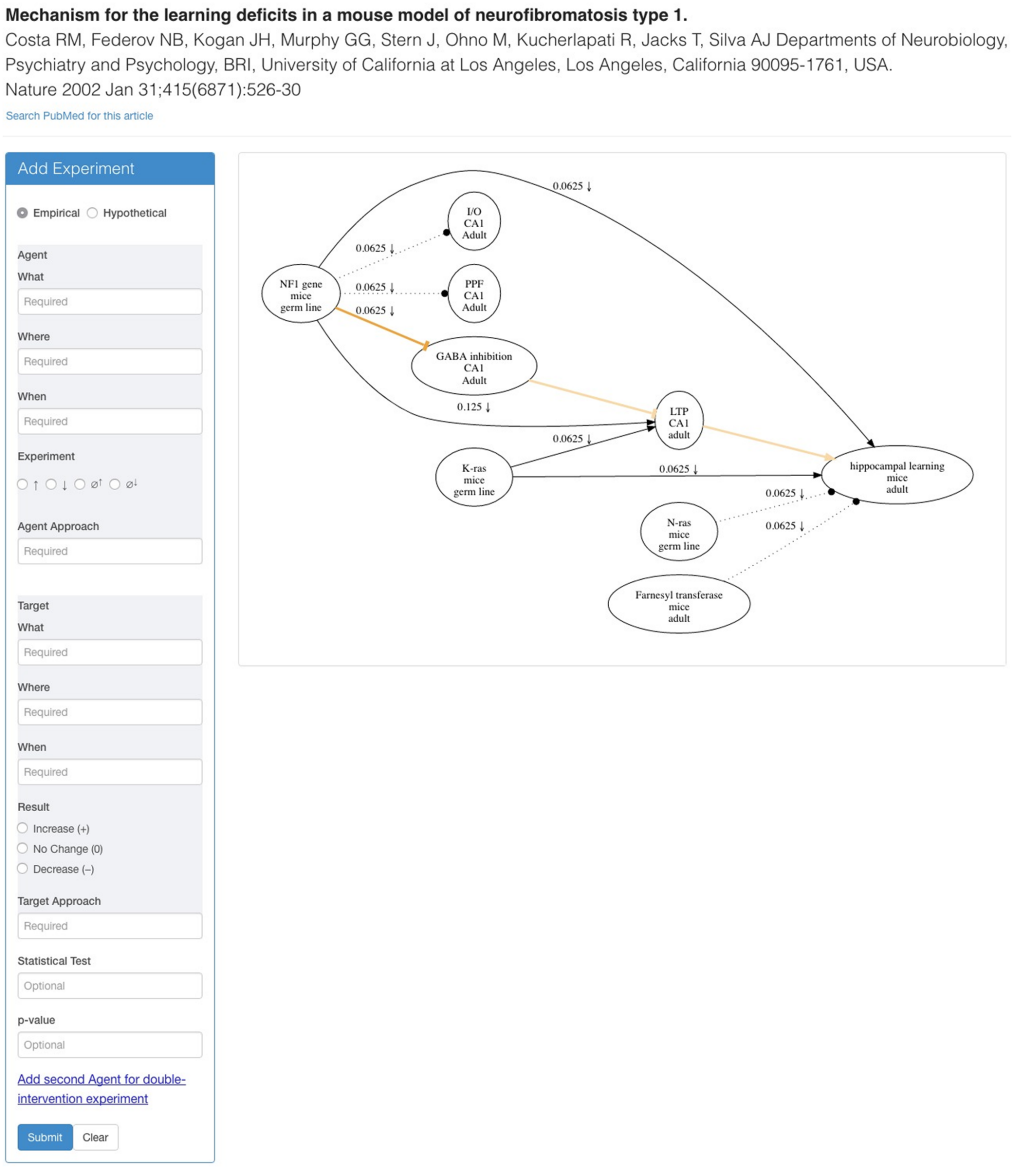
Entering information in ResearchMaps. The left panel shows the interface used to input information. The citation on the top refers to the article whose research map is displayed. Highlighted in yellow are the edges that reflect the main findings in that article. Users can double-click on any edge in the research map to retrieve PubMed citations that are potentially relevant to the edge’s Agent–Target relation.

Fig 6 shows the application’s interface for creating research maps. The fields include the What, Where, and When properties for both the Agent and the Target, the class of experiment, the type of result, and, for empirical edges, succinct descriptions of the approaches used to (1) observe or intervene on the Agent and (2) measure changes in the Target. When information is entered into the form, the research map is updated accordingly. When a research map is created for an article that is indexed on PubMed, that research map is made public to all users. However, being first and foremost a tool for the personal curation of research information, ResearchMaps can also be used to create private maps, visible only to the users who entered them. These private maps can include unpublished experiments of ongoing projects, purely speculative models, etc.

There are multiple steps to make a research map for a given research article. The first step is to identify all the nodes that will be included in the research map. This process entails the identification of Agent–Target pairs involved in the reported experiments. For any one Agent–Target pair, the next step is to find the experiment class that was performed to test their relation. In addition to the class of the experiment, the user can record the result that was obtained, as well as the key techniques that were used to observe (or manipulate) the Agent and observe the result in the Target. Once the empirical edges are entered (ones for which an experiment is reported), any hypothetical edges suggested by the article can be added, thereby helping to structure the map and contextualize the empirical results. Finally, because research maps can become large and complex, it is instructive to highlight the main connections, whether they are hypothetical or empirical.

### Combining research maps from multiple research articles

**Fig 7:**
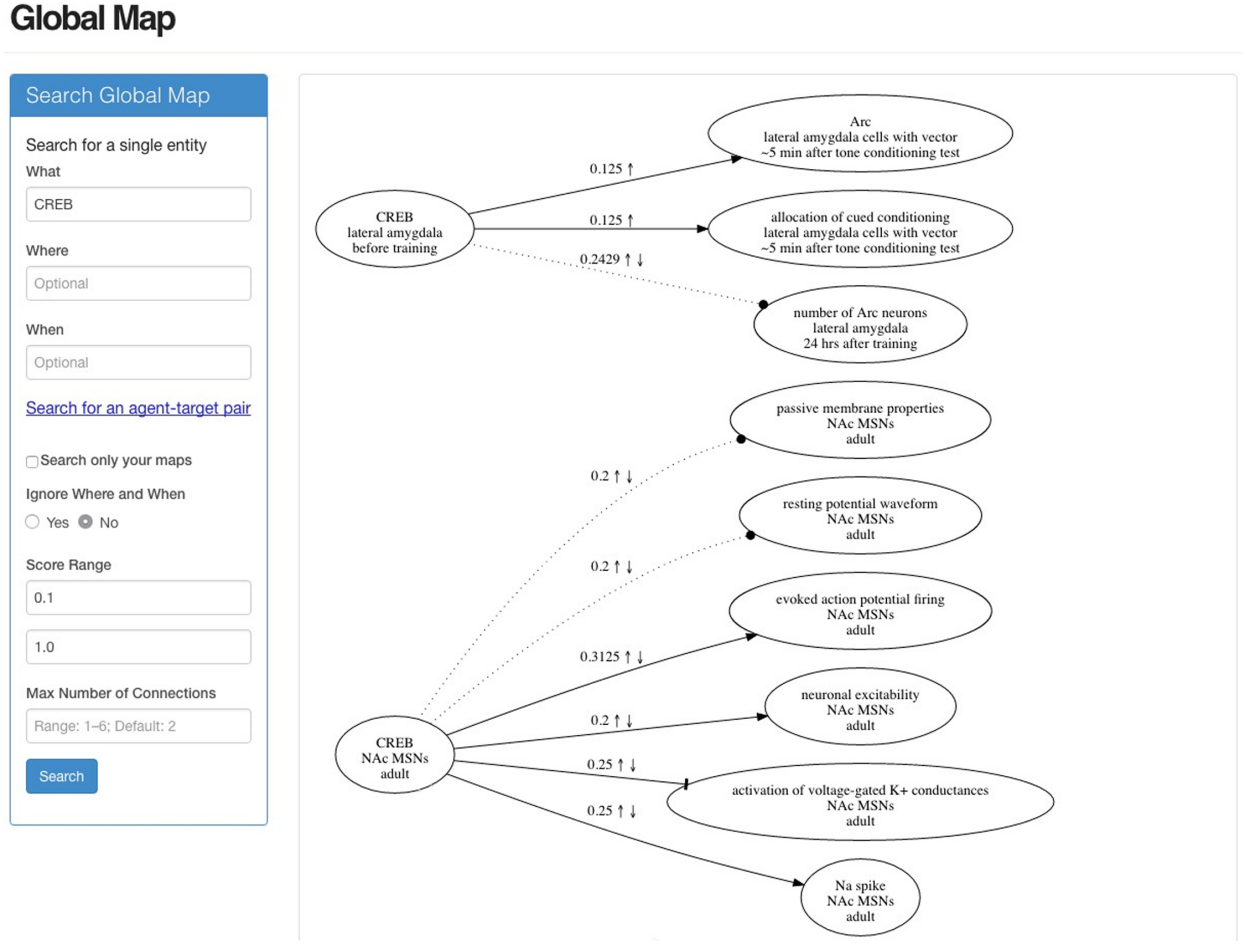
Interacting with the information in ResearchMaps. This screenshot shows the interface used to interact with the information in the app. The panel on the left is used for entering the details for a particular query (e.g., CREB). The map shown on the right includes only a fraction of the edges that this query returned. This map represents integrated data from many different research articles.

In addition to viewing the research maps of individual research articles, users can interact with all of the public data and their individual private data via the Global Map page. On this page, users can search the application’s database either for a specific node (with a What, Where, and When) or simply for a term—e.g., the transcription factor CREB (Fig 7). Additionally, users can search not only for a single entity but also for specific Agent–Target pairs, whether they are empirical or hypothetical.

To constrain the visualizations produced by queries, users can modify each global search with several parameters, including a minimum and maximum threshold for filtering empirical edges based on their scores. By filtering out edges with low scores, for example, users can visualize only those connections with the highest levels of evidence (i.e., those that are likely to be more reliable). Similarly, by filtering out edges with high scores, users can quickly identify those connections with the least amount of evidence (i.e., those in greatest need of further investigation). Users can also limit the number of edges that must be traversed between a given query term and its results. Additionally, users can limit global searches to only the information that they personally entered, thus focusing searches to specific domains of interest. This dynamic interaction with the information in ResearchMaps provides critical hypothesis-building tools, allowing users to explore the ramifications of different hypotheses.

**Fig 8:**
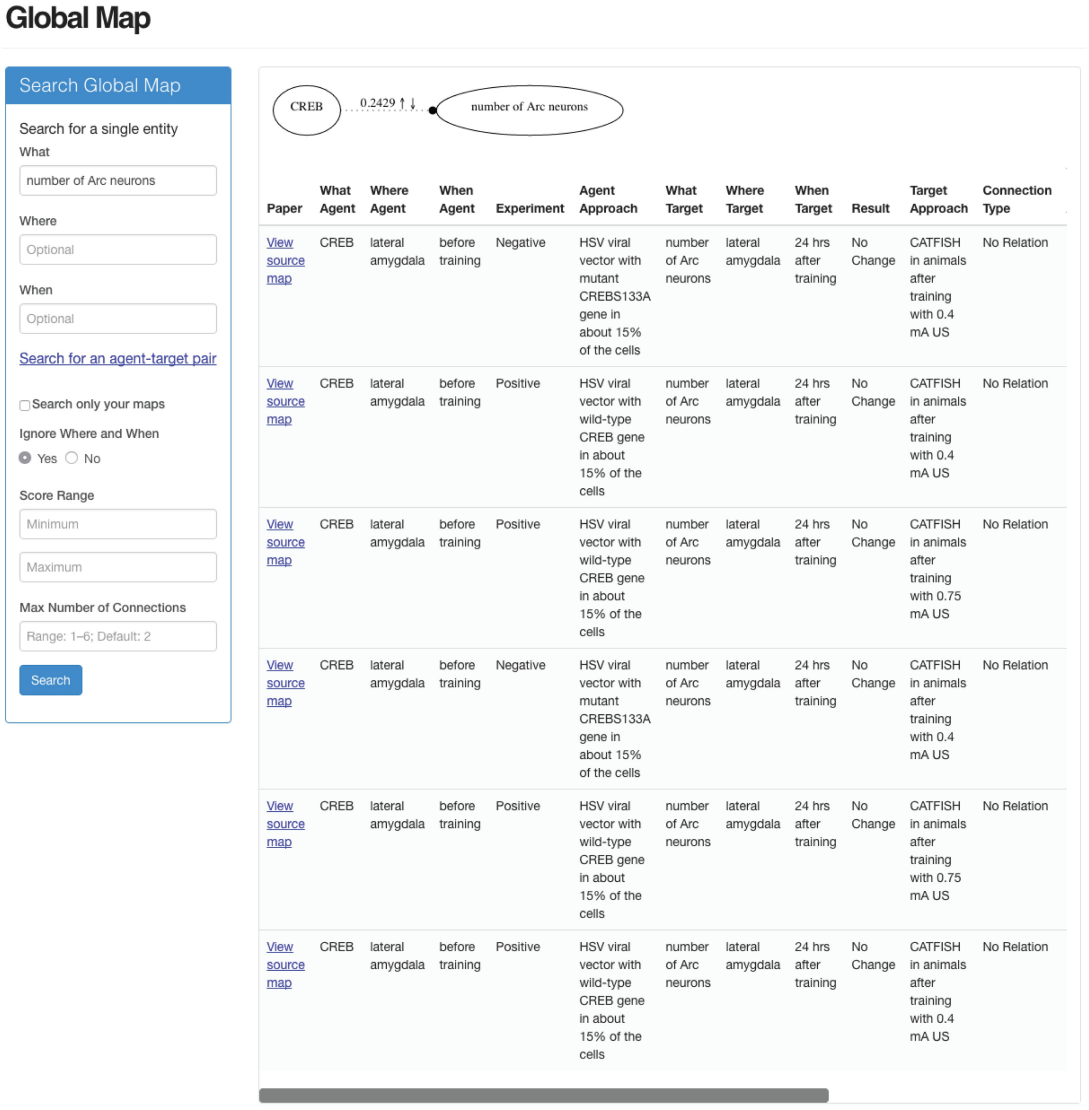
Establishing the provenance of edges in the Global Map page. The table in the bottom right of the screenshot appeared in response to clicking on the edge above the table. Hyperlinks in the left column of the table direct users to the individual research maps for each empirical and hypothetical assertion represented in that edge.

Clicking on any edge in the Global Map generates a table (see Fig 8) that lists all of the information represented by that edge. Also provided are hyperlinks that establish the provenance of any edge in the Global Map by connecting the user to the research map(s) where that edge was originally entered.

### Research maps at work

As stated above, research maps are designed to facilitate the personal curation of information derived from detailed analyses of research articles, reviews, and other sources central to the activities of scientists. The derived maps are designed to function at the interface between the large body of information that could potentially be relevant to any one individual scientist, and that subset of empirical and hypothetical assertions that an individual scientist judges to be directly relevant to ongoing work. For example, in the space of three years, one of our users created public research maps for 125 articles with 2,251 experiments, 1,293 nodes, and 1,693 edges. Even in this relatively small set of articles, the sheer number of empirical and hypothetical relations is too large for most individual scientists to remember, objectively integrate, and systematically reason through.

**Fig 9:**
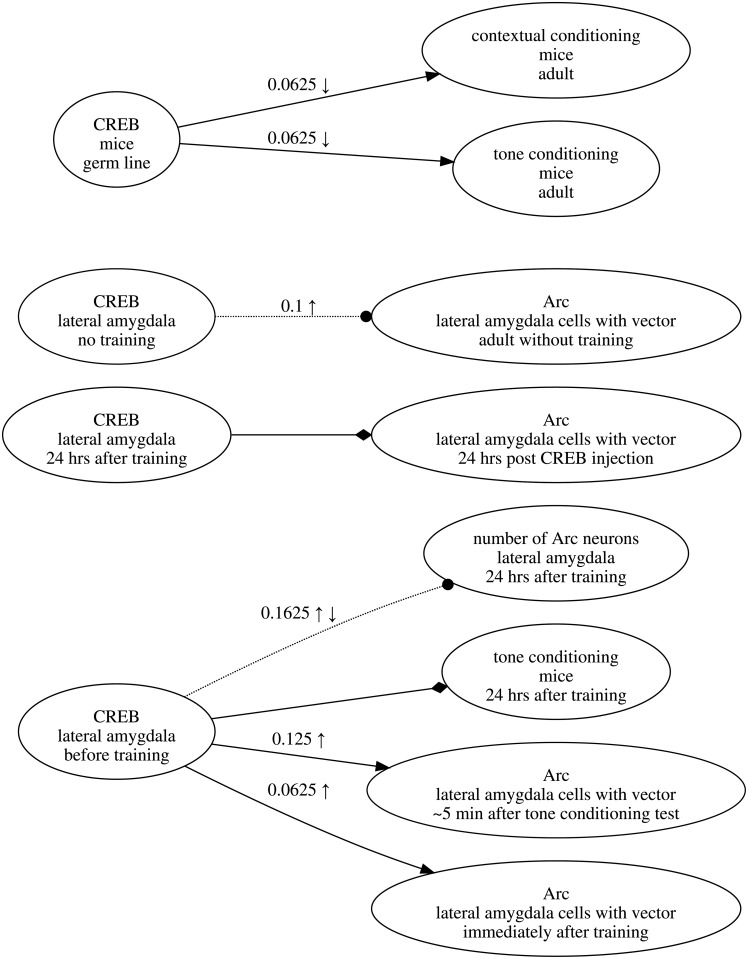
Initial map of experiments exploring the role of CREB in amygdala memory enhancements. This research map represents a series of experiments designed to explore the role of CREB expressed in a subset of lateral amygdala neurons in an enhancement of auditory and contextual conditioning.

**Fig 10:**
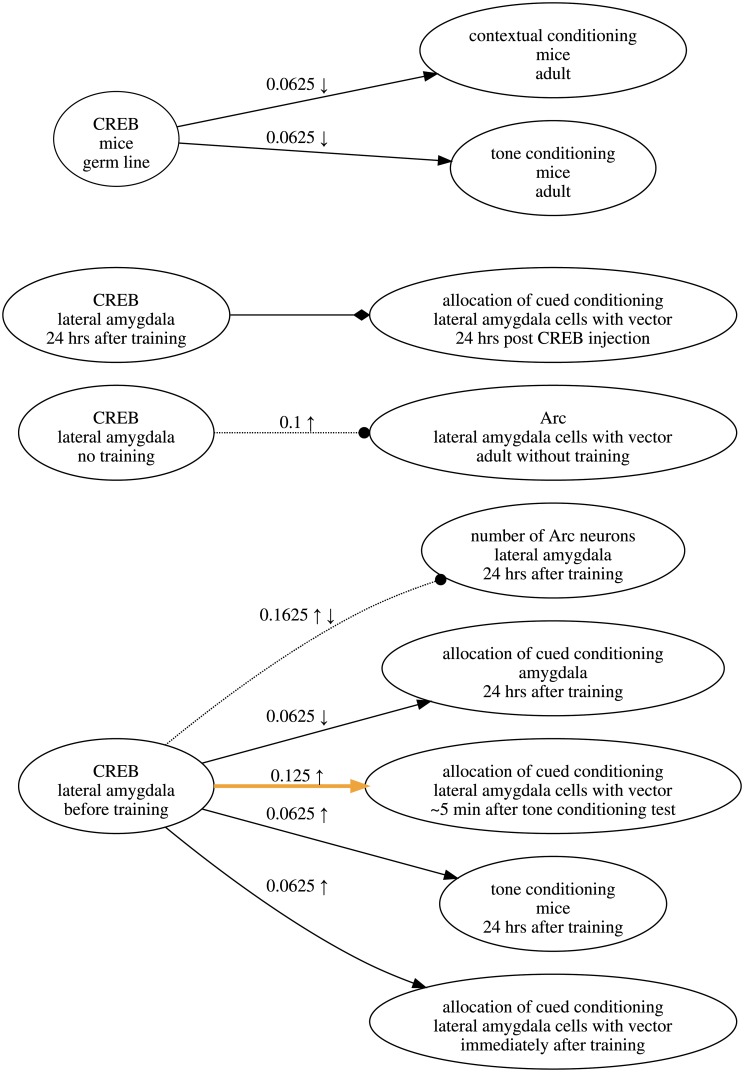
Map of experiments exploring the role of CREB in memory allocation. Inventing the concept of memory allocation with the help of research maps not only helped to structure our experiments to test the role of CREB in the amygdala during memory formation but also helped us to plan future experiments. The edge highlighted in orange points to the key experiments in the map representing early experiments on memory allocation. All the other edges represent control experiments that helped us to interpret the memory allocation experiments.

Additionally, the process of mapping information critical for a project affords a clarity that is harder to come by any other way. For instance, a few years ago some of us were involved in experiments that suggested that the expression of the cAMP responsive element binding (CREB) transcriptional factor in a small subset of neurons in the lateral amygdala of mice could lead to enhancements of memory for both auditory and contextual fear conditioning. These results were surprising, and they led to a series of experiments that explored the nature of these memory enhancements. One of the motivations for these experiments was the hypothesis that the cellular levels of CREB may be one factor that determines the subset of lateral amygdala neurons that go on to store a given fear memory [11]. The initial research map of the experiments designed to explore the CREB memory enhancement is shown in Fig 9. While thinking of the connections in that article with the help of research maps, we realized that there may be a more fundamental concept that could both provide a better structure for the map and a more useful framework for future experiments (Fig 10).

In our initial experiments [11], we used positive and negative manipulations of CREB, and determined which lateral amygdala cells were involved in memory by using the immediate early gene Arc, a gene whose expression is thought to tag cells involved in memory [12]. Mapping these findings helped us to realize that we needed to identify a phenomenon that captured the idea that CREB was instrumental in determining which cells were involved in memory. To this end, we borrowed a term from computer science—*memory allocation*—and the process of defining this new neuroscience phenomenon in our research maps also helped us to identify the need for other methods to measure it [13].

Research maps also helped us to focus our attention on the mechanisms by which CREB modulated memory allocation [13, 14] and aided us in defining a research plan to tackle this new complex problem. Although it is possible that we and others could have arrived at similar research decisions without the help of research maps, the ability to precisely map information imparted a degree of clarity that helped us to think through these experiments and develop our past and current research on memory allocation. The concept of memory allocation [15] that emerged out of these efforts led to a number of research articles [13, 14, 16–18] that explored the mechanistic basis of this concept and tested its possible role in other brain structures, such as the insular cortex [19] and in processes such as memory linking [8, 20].

When reading new research articles, the underlying mechanisms are not always apparent. However, in our experience, the process of extracting and formalizing information about possible connections tested in these articles has always enhanced our understanding of the reported findings. This formalization process also brings the information from disparate articles into a shared framework that facilitates integration of this information, as well as experiment planning.

**Fig 11:**
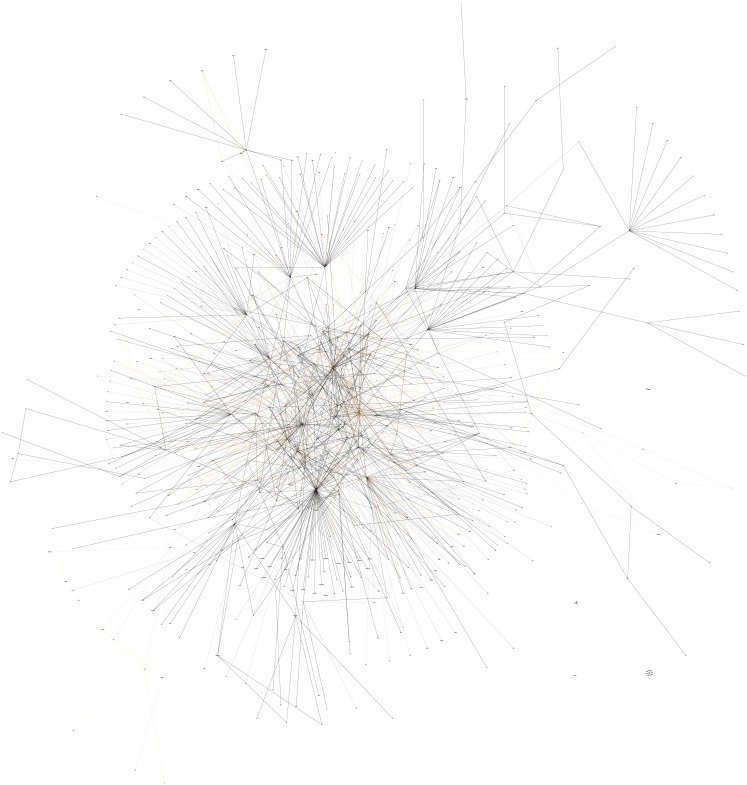
Global research map of experiments in memory allocation and other related work. This is a personally curated research map of work in the field of memory allocation and other related work that either overlaps or connects to the work in memory allocation. To minimize the number of nodes, only the What property of each node is shown, so that nodes with different Where and When properties (but identical What properties) are collapsed into one. Nodes in orange appear only in research maps for articles on memory allocation. Nodes in red appear not only in research maps for articles on memory allocation but also in research maps for related work. This research map has helped us to contextualize work in memory allocation and propose hypotheses concerning the mechanistic basis of this process.

**Fig 12:**
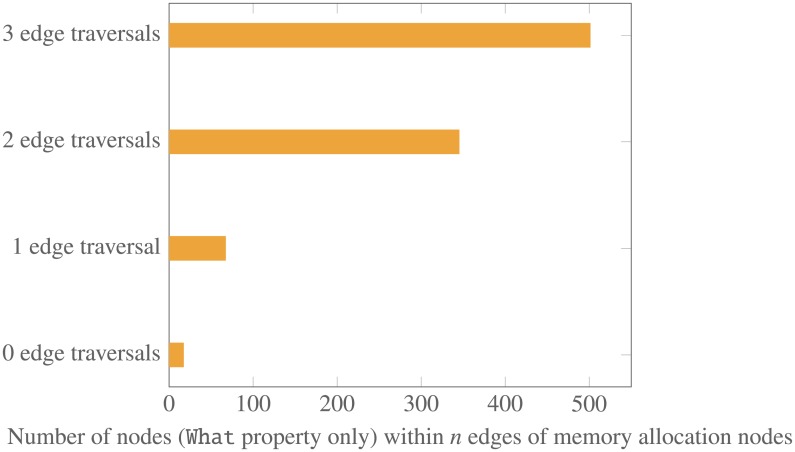
Connectivity characteristics in the Global Map for memory allocation. The plot shows the surprisingly extensive connectivity between papers in memory allocation and other related work, which can be visualized in the Global Map of ResearchMaps. For example, the graph shows that within three edge traversals, there are over 500 nodes that connect with nodes in individual memory allocation research maps. This extensive connectivity provides abundant opportunities for hypothesis building, since any one of the connected nodes could modulate unknown features of memory allocation (and vice versa).

Using research maps to visualize our work in memory allocation has also provided insight into how these experiments are connected to research in related areas. Fig 11 shows a research map of our work in memory allocation and all other research maps of articles that connect to it. Fig 12 shows a bar graph indicating the number of nodes in our ResearchMaps database that are connected to nodes pertaining to work in memory allocation. Analysis of the data represented in Figs 11 and 12 suggests that research maps provide a rich platform in which to generate and evaluate hypotheses about the mechanisms that may be modulating memory allocation.

**Fig 13:**
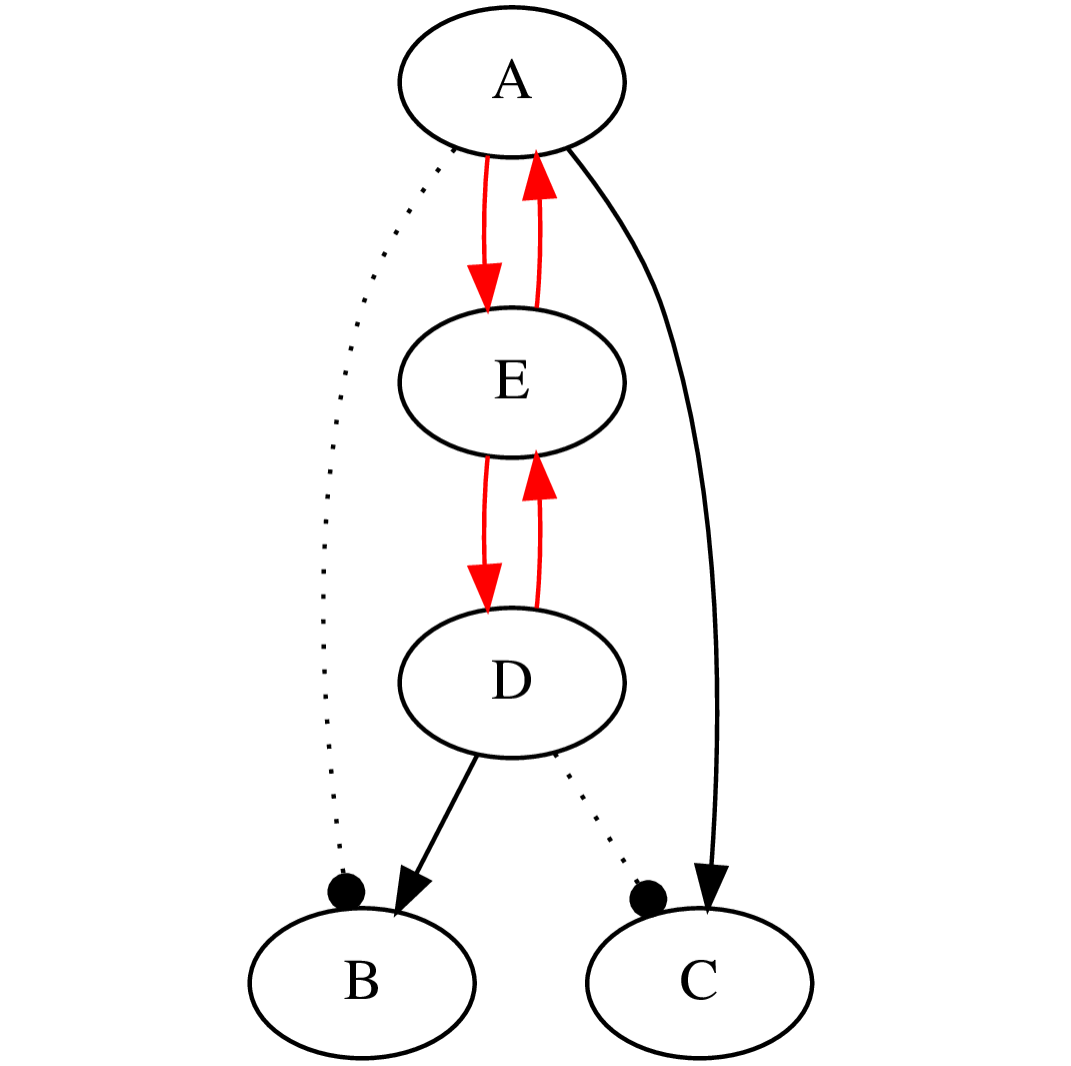
An example of how the structural information in a research map can help to identify conflicting results. The edges in red are in conflict with the edges in black—all the edges cannot be true simultaneously. (For simplicity, the scores and experiment symbols in this research map have been omitted).

In the context of evidence synthesis and experiment planning, another use case facilitated by ResearchMaps is conflict detection. The research map in Fig 13 illustrates an example in which pathways that conflict with other results are shown in red. The black edges of this research map show that the pairs (*A*, *B*) and (*C*, *D*) were found to be independent; additionally, *A* was found to excite *C*, and *D* was found to excite *B*. The red edges show the excitatory pathways *A*→*E*→*D* and *D*→*E*→*A*. Note that the edges shown in red and black cannot all be true: the excitatory pathway *A*→*E*→*D*→*B* conflicts with the finding of independence between *A* and *B*; by symmetry, the excitatory pathway *D*→*E*→*A*→*C* conflicts with the finding of independence between *C* and *D*. One insight that comes out of this analysis is that nodes *A* and *D* cannot be causes of each other—either direct or indirect. In one of the author’s research maps for a published article, exactly this conflict emerged (the biological details are omitted here for brevity), leading us to revise our interpretation of the article’s results.

We note two advantages of this approach to conflict detection. The first is that these types of analyses can be “recycled”: once an inference has been made, a database of research maps can be queried for other instances of the structural template—the specific configuration of nodes and edges—that permitted the original inference; note that the inference will hold regardless of the identity of the nodes involved. The second advantage is that combining structural information from multiple articles allows one to make inferences about biological phenomena that may have never even appeared together in the same experiment, or which were never discussed together in a single article. Although conflicts among results may be apparent if they occur in a single article, the various components of the structural template behind a conflict may be spread out across many articles, making it difficult to find. It it thus extremely difficult to anticipate where such conflicts might arise, and it is challenging to notice ones derived from the synthesis of many articles, unless one is already looking for a specific pattern—particularly as the patterns become increasingly complex.

Another use case facilitated by ResearchMaps is the collection of structured evidence for meta-analytic causal discovery—that is, the identification of causal structures that are consistent with evidence drawn from research articles [21]. Causal discovery algorithms usually take primary data as their input; less explored is the problem of generalizing these methods to meta-analytic techniques that can incorporate multiple forms of causal information, including qualitative knowledge from literature. For this approach, ResearchMaps structures causal information not only with a vocabulary that biologists recognize but also in a way that is amenable for use in constraint-based causal discovery methods (e.g., [22]).

## Discussion

Here we describe the operationalization of experimental strategies to gauge the strength of specific connections between biological phenomena. The derived algorithms and framework are implemented in ResearchMaps, a web application that generates graph-based representations (research maps) of empirical results and hypotheses. This web application enables individual biologists to track, evaluate, and systematically reason through proposed connections between biological variables that are most important to them with a breadth and precision that are unlikely without such a tool.

Beyond organizing experiments, ResearchMaps can be used to determine which additional experiments could be done to further test any connection in a research map. Thus, one of ResearchMaps’s key practical applications is to organize experimental evidence designed to test specific connections, and thereby reveal those supported by the strongest evidence. For example, knowing which specific connections have weak support and which experimental evidence could strengthen them is helpful in determining which experiments to perform next. The more systematically and explicitly we can render this knowledge, the more thorough the basis is for these choices. By making scientific interpretations of the literature explicit, research maps formalize aspects of knowledge representation and experiment planning via shareable maps that can help to derive consensus in a research community.

In addition to empirical information, ResearchMaps also provides an interface that individual biologists can use to systematically capture complex networks of hypothetical connections among biological variables, thus easing their integration. Such a tool is particularly useful for biological disciplines (e.g., neuroscience) in which individual scientists are asked to integrate information and results from multiple disparate fields. Integration in these fields is especially challenging because of the different terminologies and paradigms used; ResearchMaps meets a need for a shared framework and vocabulary for information that bridges the integrated fields. For example, it is not uncommon for articles in neuroscience to include information from fields as diverse as molecular and cellular biology, physiology, and behavioral neuroscience. Although research maps have been specifically designed to accommodate experiments in fields as diverse as molecular and cellular biology, immunology, developmental biology, and neuroscience, we imagine that future modifications to the representation will enable it to accommodate experimental designs in other fields.

Beyond curating experiments that test possible connections between variables considered in individual projects or articles, ResearchMaps allows individual researchers to integrate experiments from many different sources, thus affording the opportunity for a macro level of integration that would be difficult without it. The Global Map allows investigators to integrate not only their own maps but also the public maps contributed by the entire user base. This feature provides a rich platform for collaboration and cross-fertilization of ideas and findings.

ResearchMaps’s entries are not restricted by a formal ontology. Our current user base consists mostly of neuroscience researchers and thus, in this initial release of the application, we use the Neuroscience Information Framework’s (NIF’s) NeuroLex lexicon (http://nif-services.neuinfo.org/ontoquest/reconcile) to suggest auto-completions as users enter data. However, the lexicon in NeuroLex is limited, so we chose to allow users to enter their own terms. As the user base expands, we can envision creating an evolving ontology that facilitates sharing and integration of research maps across users [23, 24]. In principle, the nodes of a research map can refer to concepts drawn from a variety of ontologies. In future iterations of ResearchMaps, we plan to use APIs to link the What, Where, and When properties to ontological concepts, such as those of Gene Ontology [25].

The What, Where, and When properties for each node may not be sufficiently descriptive in all contexts; however, such ontological issues are well known and thus the subject of much research in computer science—we do not claim to resolve any significant challenges in this specific domain. Although the What, Where, and When properties do not address all ontological issues, they represent a pragmatic compromise between the demands placed on users and the ability to unambiguously define each node. As ResearchMaps was designed to be a personal tool, users are free to adhere to their own vocabularies and naming conventions to achieve ontological consistency across the research maps they create.

When graphical representations grow too large, their growing complexity limits their usefulness. However, ResearchMaps mitigates this issue in two key ways. First, users can choose to highlight critical edges that convey the gist of a particular research project or article. Additionally, in the Global Map, users can constrain the visualizations of the resulting maps with a variety of queries and filters—e.g., searching for specific Agent–Target pairs, filtering edges by their scores, and choosing whether to consider the Where and When properties of nodes when aggregating research maps. ResearchMaps simplifies much of the information in research articles at the expense of some key experimental details, such as a full account of the relevant background conditions of each experiment. However, this design limits the complexity of the representation, yielding a graph structure that is amenable to human exploration. ResearchMaps thus represents a compromise between detail and utility. Our representation captures what we judge to be the minimum amount of information that is essential for helping biologists to record and integrate experiments. For instance, although we use a Bayesian approach to calculate each score, the evidence calculus that we employ is meant to be representative of epistemic (not probabilistic) reasoning, and is not meant to substitute for formal causal models. The edges of a research map do not correspond to the edges in a formal causal graph (e.g., they do not differentiate between ancestral and direct relations). Instead, the edges in research maps represent experimental results in a manner familiar to many biologists and in a way that we believe may facilitate the specification of more traditional and quantitative representations of causality.

Although at this point research maps is an individual, personally curated tool, we can imagine a future where many components of this process will be automated. Natural language processing and machine learning algorithms could conceivably be used to automate the process of entering experiments into ResearchMaps. This automation would provide scientists easy access to structured information from the entire literature and would further efforts to use computers to aid and even augment the creative process in science [26]. Nevertheless, manual data entry does have its benefits. First, the user has complete freedom over which experiments and hypothetical assertions are represented, thus enabling the inclusion of only experimental and hypothetical information that they trust when integrating findings and planning experiments. Automation would limit users’ ability to judge the reliability of previously published information—a critical component of experiment planning. Although automation would provide easy access to graphical representations of Connection Experiments in the ever-growing deluge of biological information, it may distance researchers from the laboratory results that are most directly relevant to them. For example, details not captured by these graphical representations could be critical in evaluating how best to interpret and use this information in experiment planning [2].

In future work, we plan to perform usability studies with ResearchMaps to further demonstrate the utility and reproducibility of its annotation schema. To evaluate the app’s reproducibility, we will ask a group of biologists to individually annotate a corpus of research articles; we will then assess whether their annotations diverge significantly. To evaluate the app’s utility for experiment planning, we propose the following evaluation: a group of biologists will use ResearchMaps to annotate a corpus of articles published before a given cutoff date. With the research maps that the biologist create, we will use the research-map scoring approach to identify which next experiments would be most instructive. We will then assemble two lists of experiments: (1) those that were published in the literature after the cutoff date and (2) those that were identified as optimal, based on the scoring approach. The biologists, who will be blinded to the source of each experiment, will then evaluate the appropriateness of each experiment, given the work that is reported in the pre-cutoff corpus.

The principles used in research maps were developed for scientific purposes, but their usefulness is not restricted to biology or even science. The principles that govern the detection and structure of connections in science, such as convergence and consistency, are likely to be generally useful. Given that information of varying quality and dependability now spreads widely, the approaches used in research maps could be used to ferret out the potential connections in the social, political, and economic forces that shape our world. For example, maps of information inspired by research maps could be used together with other evolving strategies to advance the Semantic Web. Information, whether in science, politics, economics, or elsewhere, is likely to play by some of the same rules. Therefore, the principles behind research maps could be used to bring structure and order to the morass of confusion and contradictions that characterize our age of information.

## Methods

The choice of implementation technologies was driven by the need for the server side web application to be scalable, efficient, and cross-platform. Since the entire web application is open-source, with expected community involvement, only open-source components were used.

ResearchMaps is hosted by Amazon Web Services (AWS) Elastic Compute Cloud (EC2) (Amazon.com, Inc., Seattle, Washington, USA) with the Ubuntu 12.04 64-bit operating system. This web application is accessible at http://www.researchmaps.org, and the source code is publicly available at https://github.com/ResearchMaps/. Node.js (https://nodejs.org/) is the runtime environment responsible for client requests. The HTML is supplemented by the Bootstrap framework (http://getbootstrap.com/) for standard components, while D3.js [27] is used to modify the visualized graphs, which are created as SVG files using Graphviz [28]. Scripting is handled by JavaScript, often accompanied by the jQuery library (https://jquery.com/). We use PubMed’s interface (http://eutils.ncbi.nlm.nih.gov) to retrieve information about research articles and the NeuroLex API (http://nif-services.neuinfo.org/ontoquest/reconcile) provided by the Neuroscience Information Framework [29] to retrieve suggested auto-completions for users’ input.

ResearchMaps uses the Neo4j 2.2.1 graph database and its query language, Cypher (Neo Technology, Inc., San Mateo, CA, USA). Graph databases store data in nodes and edges (as opposed to the tuples used in relational databases) and are well-suited to our application [30]. Our Neo4j schema is designed as follows. Each user is assigned a User node, which is connected to Paper nodes that represent each research article (or private project) for which a user creates a research map. Each Paper node is connected to a number of Experiment nodes—one for each experiment (or hypothetical assertion) that is entered for a given map. Each Experiment node is connected to two NeurolexTerm nodes representing the Agent and the Target for that particular experiment. Agent and Target (NeurolexTerm) nodes are connected by edges with properties to store the information used to calculate each edge’s score.

## Supporting information

S1 Fig The Bayesian scoring approach closely resembles an earlier heuristic approach that captured scientists’ intuitions regarding empirical evidence. These plots are reproduced from Fig 3, with a second data series (in gray) showing how an earlier heuristic scoring approach [2] compares to the Bayesian one presented in this article.
(EPS)

S2 FigThe heuristic approach for calculating an evidence score, which was used in early versions of research maps.These equations were used to calculate the second data series (in gray) in S1 Fig Each cell in the table starts with a value of zero, and each empirical result increments the appropriate cell by one. The function Max(E,N,I) returns the maximum value from the set {E,N,I}. The edge’s relation is assigned according to this maximum value: either Excitatory (E), No-connection (N), or Inhibitory (I).(EPS)

S3 Fig The research map of Fig 1 with its hypothetical edges removed. This modified research map, when compared with the one in Fig 1, illustrates how hypothetical edges help to structure research maps with empirical edges, thereby augmenting the interpretation of results.
(EPS)

## References

[1] Landreth A, Silva AJ. The need for research maps to navigate published work and inform experiment planning. Neuron. 2013;79(3):411–415. doi: 10.1016/j.neuron.2013.07.024 23931992 10.1016/j.neuron.2013.07.024

[2] Silva AJ, Müller KR. The need for novel informatics tools for integrating and planning research in molecular and cellular cognition. Learning and Memory. 2015;22(9):494–498. doi: 10.1101/lm.029355.112 26286658 10.1101/lm.029355.112 PMC4561409

[3] Matiasz NJ, Wood J, Wang W, Silva AJ, Hsu W. Computer-aided experiment planning toward causal discovery in neuroscience. Frontiers in neuroinformatics. 2017;11 doi: 10.3389/fninf.2017.00012 28243197 10.3389/fninf.2017.00012 PMC5304468

[4] Russ TA, Ramakrishnan C, Hovy EH, Bota M, Burns GA. Knowledge engineering tools for reasoning with scientific observations and interpretations: a neural connectivity use case. BMC bioinformatics. 2011;12(1):351 doi: 10.1186/1471-2105-12-351 21859449 10.1186/1471-2105-12-351 PMC3176268

[5] Friedman N. Inferring cellular networks using probabilistic graphical models. Science. 2004;303(5659):799–805. doi: 10.1126/science.1094068 14764868 10.1126/science.1094068

[6] Nishimura D. BioCarta. Biotech Software & Internet Report: The Computer Software Journal for Scient. 2001;2(3):117–120. doi: 10.1089/152791601750294344

[7] Lally A, Bachi S, Barborak MA, Buchanan DW, Chu-Carroll J, Ferrucci DA, et al WatsonPaths: scenario-based question answering and inference over unstructured information. Yorktown Heights: IBM Research 2014;.

[8] Cai DJ, Aharoni D, Shuman T, Shobe J, Biane J, Song W, et al A shared neural ensemble links distinct contextual memories encoded close in time. Nature. 2016;534(7605):115 doi: 10.1038/nature17955 27251287 10.1038/nature17955 PMC5063500

[9] Silva AJ, Landreth A, Bickle J. Engineering the Next Revolution in Neuroscience: The New Science of Experiment Planning. Oxford: Oxford University Press; 2014.

[10] Spirtes P, Glymour C, Scheines R. Causation, Prediction, and Search, Second Edition. MIT Press, Cambridge, MA; 2000.

[11] Han JH, Kushner SA, Yiu AP, Cole CJ, Matynia A, Brown RA, et al Neuronal competition and selection during memory formation. science. 2007;316(5823):457–460. doi: 10.1126/science.1139438 17446403 10.1126/science.1139438

[12] Guzowski JF, McNaughton BL, Barnes CA, Worley PF. Environment-specific expression of the immediate-early gene Arc in hippocampal neuronal ensembles. Nature neuroscience. 1999;2(12). doi: 10.1038/16046 10570490 10.1038/16046

[13] Zhou Y, Won J, Karlsson MG, Zhou M, Rogerson T, et al CREB regulates excitability and the allocation of memory to subsets of neurons in the amygdala. Nature neuroscience. 2009;12(11):1438 doi: 10.1038/nn.2405 19783993 10.1038/nn.2405 PMC2783698

[14] Han JH, Kushner SA, Yiu AP, Hsiang HLL, Buch T, Waisman A, et al Selective erasure of a fear memory. Science. 2009;323(5920):1492–1496. doi: 10.1126/science.1164139 19286560 10.1126/science.1164139

[15] Frankland PW, Josselyn SA. Memory allocation. Neuropsychopharmacology. 2015;40(1):243 doi: 10.1038/npp.2014.234 25482172 10.1038/npp.2014.234 PMC4262914

[16] Sargin D, Mercaldo V, Yiu AP, Higgs G, Han JH, Frankland PW, et al CREB regulates spine density of lateral amygdala neurons: implications for memory allocation. Frontiers in behavioral neuroscience. 2013;7 doi: 10.3389/fnbeh.2013.00209 24391565 10.3389/fnbeh.2013.00209 PMC3868910

[17] Kim J, Kwon JT, Kim HS, Josselyn SA, Han JH. Memory recall and modifications by activating neurons with elevated CREB. Nature neuroscience. 2014;17(1):65–72. doi: 10.1038/nn.3592 24212670 10.1038/nn.3592

[18] Yiu AP, Mercaldo V, Yan C, Richards B, Rashid AJ, Hsiang HLL, et al Neurons are recruited to a memory trace based on relative neuronal excitability immediately before training. Neuron. 2014;83(3):722–735. doi: 10.1016/j.neuron.2014.07.017 25102562 10.1016/j.neuron.2014.07.017

[19] Sano Y, Shobe JL, Zhou M, Huang S, Shuman T, Cai DJ, et al CREB regulates memory allocation in the insular cortex. Current Biology. 2014;24(23):2833–2837. doi: 10.1016/j.cub.2014.10.018 25454591 10.1016/j.cub.2014.10.018 PMC4743759

[20] Rashid AJ, Yan C, Mercaldo V, Hsiang HLL, Park S, Cole CJ, et al Competition between engrams influences fear memory formation and recall. Science. 2016;353(6297):383–387. doi: 10.1126/science.aaf0594 27463673 10.1126/science.aaf0594 PMC6737336

[21] Matiasz NJ, Wood J, Wang W, Silva AJ, Hsu W. Translating literature into causal graphs: Toward automated experiment selection. In: Proceedings of the IEEE International Conference on Bioinformatics and Biomedicine (IEEE BIBM); 2017.

[22] Hyttinen A, Eberhardt F, Järvisalo M. Constraint-based Causal Discovery: Conflict Resolution with Answer Set Programming. In: Proceedings of the 30th Conference on Uncertainty in Artificial Intelligence (UAI 2014). Quebec City, Quebec; 2014. p. 340–349.

[23] Flouris G, Plexousakis D, Antoniou G. Evolving ontology evolution. In: SOFSEM. vol. 3831. Springer; 2006. p. 14–29.

[24] Kondylakis H, Plexousakis D. Exelixis: evolving ontology-based data integration system. In: Proceedings of the 2011 ACM SIGMOD International Conference on Management of data. ACM; 2011. p. 1283–1286.

[25] Ashburner M, Ball CA, Blake JA, Botstein D, Butler H, Cherry JM, et al Gene Ontology: tool for the unification of biology. Nature Genetics. 2000;25(1):25–29. doi: 10.1038/75556 10802651 10.1038/75556 PMC3037419

[26] King RD, Rowland J, Oliver SG, Young M, Aubrey W, Byrne E, et al The automation of science. Science. 2009;324(5923):85–89. doi: 10.1126/science.1165620 19342587 10.1126/science.1165620

[27] Bostock M, Ogievetsky V, Heer J. D^3^: Data-Driven Documents. IEEE Transactions on Visualization and Computer Graphics. 2011;17(12):2301–2309. doi: 10.1109/TVCG.2011.185 22034350 10.1109/TVCG.2011.185

[28] Ellson J, Gansner E, Koutsofios L, North SC, Woodhull G. In: Mutzel P, Jünger M, Leipert S, editors. Graphviz—Open Source Graph Drawing Tools. Berlin, Heidelberg: Springer Berlin Heidelberg; 2002. p. 483–484. Available from: http://dx.doi.org/10.1007/3-540-45848-4_57.

[29] Gardner D, Akil H, Ascoli GA, Bowden DM, Bug W, Donohue DE, et al The neuroscience information framework: a data and knowledge environment for neuroscience. Neuroinformatics. 2008;6(3):149–160. doi: 10.1007/s12021-008-9024-z 18946742 10.1007/s12021-008-9024-z PMC2661130

[30] Lysenko A, Roznovăţ IA, Saqi M, Mazein A, Rawlings CJ, Auffray C. Representing and querying disease networks using graph databases. BioData mining. 2016;9(1):23 doi: 10.1186/s13040-016-0102-8 27462371 10.1186/s13040-016-0102-8 PMC4960687

